# *Streptococcus pneumoniae* Serotypes and Mortality in Adults and Adolescents in South Africa: Analysis of National Surveillance Data, 2003 - 2008

**DOI:** 10.1371/journal.pone.0140185

**Published:** 2015-10-13

**Authors:** Cheryl Cohen, Nireshni Naidoo, Susan Meiring, Linda de Gouveia, Claire von Mollendorf, Sibongile Walaza, Preneshni Naicker, Shabir A. Madhi, Charles Feldman, Keith P. Klugman, Halima Dawood, Anne von Gottberg, GERMS-SA

**Affiliations:** 1 Centre for Respiratory Diseases and Meningitis, National Institute for Communicable Diseases (NICD) of the National Health Laboratory Service (NHLS), Johannesburg, South Africa; 2 School of Public Health, Faculty of Health Sciences, University of the Witwatersrand, Johannesburg South Africa; 3 Division of Medical Microbiology, University of Cape Town, Cape Town, South Africa; 4 Department of Internal Medicine, Faculty of Health Sciences, University of the Witwatersrand, Johannesburg South Africa; 5 Hubert School of Public Health, Emory University, Atlanta, GA, United States of America; 6 Department of Internal Medicine, Faculty of Health Sciences, University of KwaZulu-Natal, Pietermaritzburg, South Africa; 7 School of Pathology, Faculty of Health Sciences, University of the Witwatersrand, Johannesburg South Africa; Faculdade de Medicina de Lisboa, PORTUGAL

## Abstract

**Background:**

An association between pneumococcal serotypes and mortality has been suggested. We aimed to investigate this among individuals aged ≥15 years with invasive pneumococcal disease (IPD) in South Africa.

**Methods:**

IPD cases were identified through national laboratory-based surveillance at 25 sites, pre-pneumococcal conjugate vaccine (PCV) introduction, from 2003–2008. We assessed the association between the 20 commonest serotypes and in-hospital mortality using logistic regression with serotype 4 (the third commonest serotype with intermediate case-fatality ratio (CFR)) as referent.

**Results:**

Among 3953 IPD cases, CFR was 55% (641/1166) for meningitis and 23% (576/2484) for bacteremia (p<0.001). Serotype 19F had the highest CFR (48%, 100/207), followed by serotype 23F (39%, 99/252) and serotype 1 (38%, 246/651). On multivariable analysis, factors independently associated with mortality included serotype 1 (OR 1.9, 95%CI 1.1–3.5) and 19F (OR 2.9, 95%CI 1.4–6.1) vs. serotype 4; increasing age (25–44 years, OR 1.8, 95%CI 1.0–3.0; 45–64 years, OR 3.6, 95%CI 2.0–6.4; ≥65 years, OR 5.2, 95%CI 1.9–14.1; vs. 15–24 years); meningitis (OR 4.1, 95%CI 3.0–5.6) vs. bacteremic pneumonia; and HIV infection (OR1.7, 95%CI 1.0–2.8). On stratified multivariate analysis, serotype 19F was associated with increased mortality amongst bacteremic pneumococcal pneumonia cases, while no serotype was associated with increased mortality in meningitis cases.

**Conclusion:**

Mortality was increased in HIV-infected individuals, which may be reduced by increased antiretroviral therapy availability. Serotypes associated with increased mortality are included in the 10-and-13-valent PCV and may become less common in adults due to indirect effects following routine infant immunization.

## Introduction

*Streptococcus pneumoniae* is a common cause of pneumonia, meningitis and septicemia and is associated with substantial morbidity and mortality worldwide[1, 2]. Since the introduction of pneumococcal conjugate vaccines (PCVs), a decline in the incidence of vaccine-serotype invasive pneumococcal disease (IPD) has been observed in vaccinated children and unvaccinated adults (through herd effect) in areas where the vaccine is widely used[3]. A significant burden of IPD, however, exists worldwide due to serotypes that are not included in PCV, which has increased in children and adults since the introduction of PCV into childhood immunization programs[4].

Incidence, severity and mortality of IPD are influenced by host- and organism-related factors. The host-related factors include extremes of age, underlying chronic illness, immunosuppression and access to antibiotic treatment[5–8]. Pathogen-related factors include the polysaccharide capsule which is the serotype determinant[9, 10]. Previous studies have shown an association between pneumococcal serotypes and mortality in adults[11, 12]. These associations could differ in a middle-income country, such as South Africa, where there is a high HIV prevalence (19% in adults aged 15–49 years in 2012)[13, 14] and a greater diversity of serotypes associated with IPD[15]. In addition, few studies have examined the association between serotypes and mortality in the absence of potential effects of pneumococcal vaccination and none have examined the association between serotype and mortality separately in HIV-infected and HIV-uninfected individuals[12].

We aimed to determine the association between pneumococcal serotype and in-hospital mortality among patients aged ≥15 years with IPD in South Africa during the period 2003–2008, prior to the introduction of PCV in the routine immunization program.

## Methods

### Surveillance for IPD

From 2003, national, active, population-based, laboratory-based surveillance for IPD was conducted through the GERMS-SA programme [16]. Reports of laboratory-confirmed IPD, including demographic details as well as date of specimen, and source of isolate together with isolates were sent to the National Institute for Communicable Diseases (NICD) in Johannesburg from >130 laboratories nationally. We conducted annual laboratory audits of all public-sector laboratories in 8 provinces annually using Disa*Lab Laboratory Information Management System. We included cases identified by audit. At 25 sentinel enhanced surveillance hospitals in all nine provinces we collected additional information from patient interviews including HIV serological status, admission date, discharge diagnosis and outcome.

Cases were defined as patients ≥15 years of age with *S*. *pneumoniae* cultured from normally sterile site specimens (e.g., cerebrospinal fluid [CSF], blood, pleural, peritoneal or joint fluid) from January 2003 through December 2008. We excluded repeat isolates from the same individual within 21 days of the initial positive culture. We defined specimen source using a hierarchical definition, as follows: CSF specimen regardless of other specimens; blood specimen regardless of other specimens (excluding CSF); and other e.g., pleural fluid, joint fluid etc. without CSF or blood. We only obtained information on clinical syndrome for enhanced surveillance sites and we defined clinical syndrome using a hierarchical definition, as follows: meningitis if a clinical diagnosis of meningitis was noted in the medical records or the pneumococcus was isolated from CSF; bacteremic pneumonia if the clinical diagnosis of pneumonia was noted in the medical records and the pneumococcus was isolated from blood culture; other (including any diagnoses not including the preceding two, including bacteremia without any localising site and localized pneumonia with pneumococcus isolated from other sterile sites only). We defined predisposing conditions other than HIV infection as follows: asplenia, sickle cell anemia; chronic illnesses including lung, liver, renal, cardiac disease and diabetes; other immunocompromising conditions including malignancy, primary immunodeficiency, immunotherapy and organ transplant; as well as other Advisory Committee on Immunization Practices [17] risk factors including head injury with CSF leak, alcohol and smoking. Disease severity was measured using the Pitt bacteremia score, which is a composite severity score including patient temperature, presence of hypotension, receipt of mechanical ventilation, cardiac arrest and mental status[18, 19]. We defined acute severe illness as a Pitt bacteremia score of ≥4 at the time of specimen collection. Provinces were grouped into 3 categories based on poverty rates (low, intermediate and high) determined by the findings of the living conditions survey conducted by Statistics South Africa in 2008–2009[20].

### Serotyping and susceptibility testing

The Quellung reaction using specific antisera (Statens Serum Institut, Copenhagen, Denmark) was used for pneumococcal serotyping. We separately distinguished serotypes 6A, B, C and D for the whole study period, although serotypes 6C and 6D were identified from <1% of cases[21]. Isolates were screened for penicillin resistance using oxacillin disc diffusion (Mast Diagnostics, Merseyside, United Kingdom) [22] and minimum inhibitory concentrations (MICs) of the potentially resistant isolates were determined using agar dilution or Etest^®^ (AB-Biodisk, Solna, Sweden). We interpreted results as non-susceptible (intermediately resistant and resistant) or susceptible using 2008 Clinical and Laboratory Standards Institute (CLSI) definitions[22]. We considered isolates with MICs ≥0.12mg/L to be non-susceptible to penicillin at using the oral penicillin breakpoint. We defined multi-drug resistance as non-susceptibility to any three or more different antibiotic classes, according to the 2009 definitions of the CLSI. [22] For determination of appropriate antibiotic prescription, data on the antibiotics used for patient management was compared with the recommended antibiotic guidelines to determine whether the appropriate antibiotic was administered to the patient[23].

### Statistical analysis

The study population included all patients with IPD aged ≥15 years from 2003 to 2008. Because our main focus was on the association between serotypes and in-hospital outcome, we included only patients with known in-hospital outcome at enhanced surveillance sites and presenting with one of the 20 most common serotypes, to allow sufficient numbers in each group for comparison. We *a priori* included the serotypes in PCV-13 i.e 1, 3, 4, 5, 6A, 6B, 7F, 9V, 14, 18C, 19A, 19F and 23F. In addition we included serotypes 8, 12F, 16, 9N, 22F, 25 and 13.

Mortality was defined as death in hospital and within 30 days of the IPD episode, to avoid inclusion of deaths unrelated to IPD. To explore possible bias introduced by including only patients presenting to enhanced surveillance sites we compared the characteristics of patients presenting to enhanced and non-enhanced sites using logistic regression.

To assess differences between serotypes we evaluated the association of age (age group 15–24 years, 25–44 years, 45–64 years, ≥65 years), clinical syndrome (meningitis, bacteremic pneumonia and other IPD), HIV co-infection and in-hospital mortality for each serotype compared to serotype 4. We chose serotype 4 as the referent as it was the third most common serotype (289/3953, 7%) identified and we wanted to use a serotype with an intermediate CFR (29%) as referent to allow for identification of serotypes with particularly high or low CFR. We used univariate multinomial regression models, generating a separate estimate of effect for each predictor on each outcome relative to the base level. The effect measures are the ratios of two relative risks (relative risk ratios) with each relative risk describing the probability of the outcome in the category of interest relative to the baseline category[24].

To determine factors associated with in-hospital mortality, we used logistic regression. We assessed all variables that were significant at p<0.2 on univariate analysis, and eliminated from the model non-significant factors (p≥0.05) with stepwise backward selection. Patients with missing data for included variables were excluded from the model. Serotype was retained in all multivariate models *a priori* because it was the main variable of interest and clinical syndrome because it is an important potential confounder. For the analysis of factors associated with mortality in HIV-infected individuals CD4 + T cell count was not included in the multivariable model because of a large amount (>40%) of missing data, however, CD4+ T cell count was not associated with serotype. All analyses were done using Stata Version 13 (StataCorp Limited). Two-sided p values <0.05 were considered significant. We performed stratified analyses for meningitis and bacteremic pneumonia separately and HIV-infected and—uninfected individuals separately, to explore whether the association between serotype and mortality varied within these subgroups. For each analysis, we used all available case information. Variables were binary (yes/no), defined as the presence or absence of the attribute excluding missing data, or categorical variables in multiple levels. We performed a sensitivity analysis restricting the study population to individuals aged ≥18 years.

### Ethics Procedures

The national surveillance programme includes national laboratory-based surveillance where demographic data and isolates are sent to NICD routinely without requiring patient consent as part of the NICD’s national public health surveillance responsibility. In addition, at sentinel enhanced surveillance sites, additional data is obtained from patient interviews, only from individuals who provide written informed consent. For participants under the age of 18, written informed consent was obtained from parents or legal guardians. The surveillance protocol (laboratory-based and enhanced surveillance) was approved by the University of the Witwatersrand Human Research Ethics Committee (Medical) (M0801117) and other relevant institutional ethics committees.

## Results

**Fig 1:**
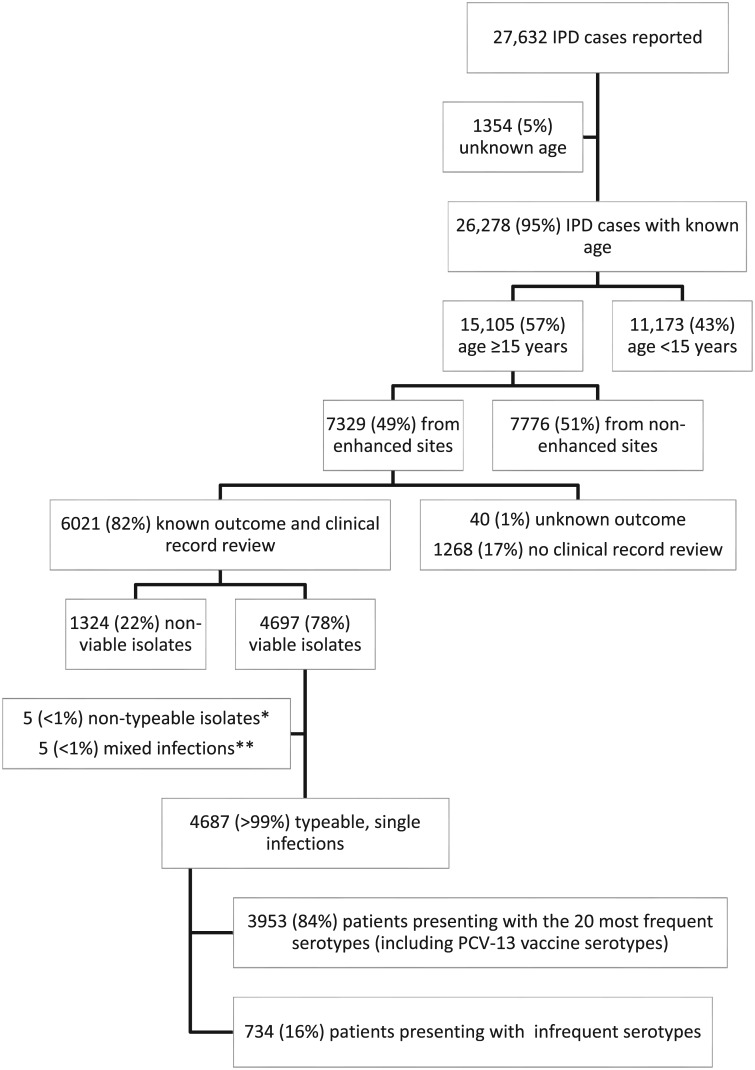
Flow diagram of patients with invasive pneumococcal disease (IPD) in South Africa, 2003–2008 and included in the analysis. *Pneumococci that did not have a capsule and could not be serotyped. **Isolates from patients that were simultaneously infected with two strains of pneumococci with different serotypes.

From 2003–2008, 27,632 patients with IPD were reported to the GERMS-SA surveillance program (Fig 1). Among 26,278 (95%) with known age, 15,105 (57%) were aged ≥15 years. Among these, 49% (7329) were from enhanced sites and 82% (6021) of these had known in-hospital outcome. Of patients with known outcomes, 4697 (78%) had viable, serotypeable isolates from a single IPD episode and 3953 (84%) of these were due to the 20 most frequent serotypes including PCV-13 vaccine serotypes. Data on clinical diagnosis was available for 99% (3923/3953) of individuals.

The overall case-fatality ratio (CFR) was 34% (1333/3953): 55% (641/1166) amongst patients with meningitis, 23% (576/2484) in patients with bacteremic pneumonia and 36% (98/273) in patients with other IPD. Serotype 1 was the commonest serotype identified (651/3953, 16%), followed by serotype 19A (443/3953, 11%), serotype 4 (289/3953, 7%) and serotype 3 (284/3953, 7%). The majority of patients (64%, 2527/3953) were aged 25–44 years, 54% (2146/3952) were female and of those with known status, 89% (2309/2580) were HIV infected.

Comparing patients at enhanced to non-enhanced surveillance sites, on multivariable analysis, patients from enhanced sites were more likely to come from an intermediate poverty level province (odds ratio [OR] 1.3, 95% confidence interval [CI] 1.2-1.5) and less likely to come from a high poverty level province (OR 0.5, 95% CI 0.4-0.6)(vs low poverty province), more likely to be of non-black race (OR 3.3, 95% CI 2.5-4.2), more likely to have blood (OR 2.4, 95% CI 2.1–2.7) or other (OR 1.3, 95% CI 1.0–1.5) specimen type (vs CSF) and more likely to have disease due to serotype 9N (OR 1.6, 95% CI 1.1–2.5) or 22F (OR 22.1, 95% CI 5.3–91.6)(vs serotype 4) (Table 1).

**Table 1:** Comparison of demographic and clinical characteristics of patients aged ≥15 years with invasive pneumococcal disease (IPD) in South Africa from GERMS-SA enhanced and non-enhanced sites, 2003–2008.

Variable		Enhanced sites	Non-enhanced sites	Univariate analysis		Multivariable analysis	
		n (%)	n (%)	OR (95% CI)	p	OR (95% CI)	p
Province*	Low poverty	5377/7329 (73)	4601/7776 (59)	Reference	Reference	Reference	Reference
	Intermediate poverty	1431/7329 (20)	1539/7776 (20)	0.8 (0.7–0.9)	<0.001	1.3 (1.2–1.5)	<0.001
	High poverty	521/7329 (7)	1636/7776 (21)	0.27 (0.3–0.3)	<0.001	0.5 (0.4–0.6)	<0.001
Race	Non-black	539/6779 (8)	93/4242 (2)	3.9 (3.1–4.8)	<0.001	3.3 (2.5–4.2)	<0.001
Specimen	CSF	1727/7329 (24)	3398/7776 (44)	Reference	Reference	Reference	Reference
	Blood	4955/7329 (67)	3161/7776 (41)	3.1 (2.9–3.3)	<0.001	2.4 (2.1–2.7)	<0.001
	Other	647/7329 (9)	1217/7776 (16)	1.1(0.9–1.2)	0.021	1.3 (1.0–1.5)	0.007
Serotype	1	463/4528 (17)	920/4434 (21)	0.9 (0.8–1.1)	0.532	0.9 (0.8–1.2)	0.541
	19A	486/4528 (11)	401/4434 (9)	1.4 (1.1–1.7)	0.002	1.06 (0.8–1.3)	0.332
	4	323/4528 (7)	368/4434 (8)	Reference	Reference	Reference	Reference
	3	323/4528 (7)	251/4434 (6)	1.5 (1.2–1.8)	0.001	1.1 (0.8–1.4)	0.559
	6A	300/4528 (7)	321/4434 (7)	1.1 (0.9–1.3)	0.751	1.0 (0.8–1.3)	0.515
	14	284/4528 (6)	282/4434 (6)	1.1 (0.9–1.4)	0.374	1.05 (0.7–1.2)	0.516
	23F	301/4528 (6)	285/4434 (7)	1.2 (0.9–1.4)	0.761	1.3 (1.0–1.7)	0.031
	8	230/4528 (5)	243/4434 (5)	1.1 (0.9–1.4)	0.968	1.0 (0.7–1.3)	0.933
	19F	240/4528 (5)	200/4434 (5)	1.4 (1.1–1.7)	0.011	1.3 (0.9–1.7)	0.079
	6B	230/4528 (5)	234/4434 (6)	1.1 (0.8–1.3)	0.571	1.0 (0.8–1.3)	0.702
	12F	215/4528 (5)	257/4434 (6)	1.0 (0.8–1.2)	0.203	0.9 (0.7–1.3)	0.858
	9V	163/4528 (4)	157/4434 (4)	1.18 (0.9–1.5)	0.708	1.3 (0.9–1.7)	0.431
	16	116/4528 (3)	108/4434 (2)	1.2 (0.9–1.6)	0.588	1.1 (0.7–1.6)	0.583
	9N	101/4528 (2)	68/4434 (2)	1.7 (1.2–2.4)	0.003	1.6 (1.1–2.5)	0.029
	7F	80/4528 (2)	67/4434 (2)	1.4 (0.9–2.0)	0.067	1.1 (0.7–1.6)	0.462
	18C	81/4528 (2)	89/4434 (2)	1.0 (0.7–1.4)	0.583	1.1 (0.7–1.6)	0.820
	25	74/4528 (2)	52/4434 (1)	1.6 (1.1–2.4)	0.014	1.1 (0.7–1.7)	0.401
	22F	74/4528 (2)	2/4434 (0.1)	42.2 (10.3–173.1)	<0.001	22.1 (5.3–91.6)	<0.001
	13	78/4528 (2)	65/4434 (1)	1.4 (1.0–2.0)	0.173	1.4 (0.9–2.1)	0.151
	5	64/4528 (1)	64/4434 (1)	1.1 (0.8–1.6)	0.944	0.9 (0.6–1.5)	0.656

N-number, OR—Odds ratio, CI—confidence interval, CSF- cerebrospinal fluid. Only factors statistically significant on univariate analysis are included in the table. Additional variables evaluated were age group and gender.

*The low poverty rate group consisted of Gauteng and Western Cape. The intermediate poverty rate group consisted of KwaZulu-Natal, Free State, Northern Cape and North West. The high poverty rate group consisted of Eastern Cape, Mpumalanga and Limpopo.

### Univariate multinomial analysis of the 20 commonest serotypes by age group, syndrome, in-hospital outcome and HIV co-infection

**Fig 2:**
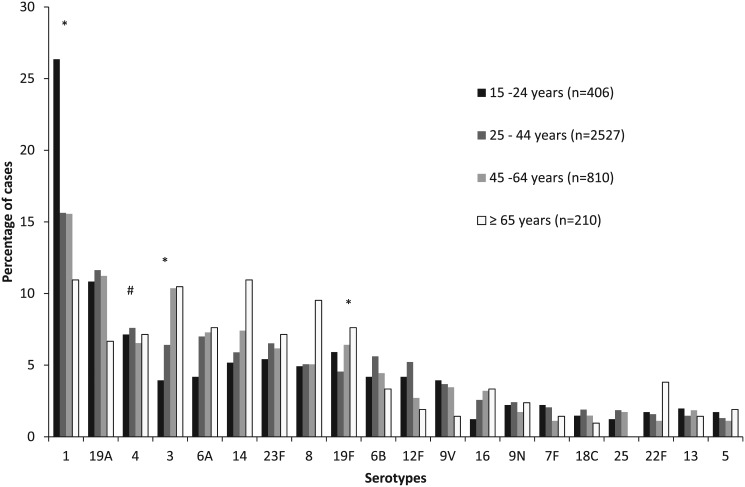
Distribution of pneumococcal serotypes amongst patients aged ≥15 years with invasive pneumococcal disease (IPD) in South Africa from 2003–2008, by age group. # Reference group. *Statistically significant at p<0.05.

Analyzing the 20 most common serotypes, using multinomial regression, comparing the age distribution of all other serotypes to the age distribution of serotype 4 (25–44 years as the referent group), serotype 3 was significantly more likely to be identified from patients 45–64 years of age (relative risk ratio [RRR] 1.9, 95% CI 1.2–2.8), serotype 1 was significantly more likely to be isolated from patients 15–24 years of age (RRR 1.8. 95% CI 1.2–2.8) and serotype 19F was significantly more likely to be isolated from patients 45–64 years of age (RRR 1.6 95% CI 1.1–2. 6) (Fig 2).

**Fig 3:**
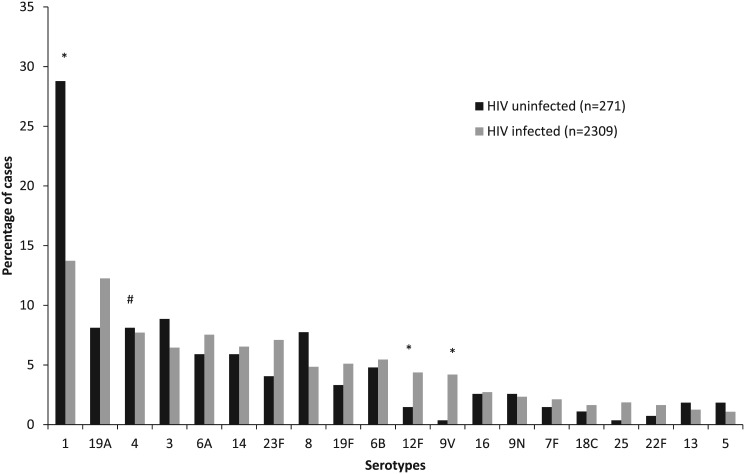
Distribution of pneumococcal serotypes amongst patients aged ≥15 years with invasive pneumococcal disease (IPD) in South Africa from 2003–2008, by HIV-infection status. # Reference group. *Statistically significant at p<0.05.

Compared to serotype 4, serotypes 12F (RRR 3.1, 95% CI 1.1–9.3) and 9V (RRR 12.0, 95% CI 1.6–90.3) caused significantly more disease in HIV-infected individuals, whereas serotype 1 (RRR 0.5, 95% CI 0.3–0.8) was significantly less likely in HIV-infected patients (Fig 3).

**Fig 4:**
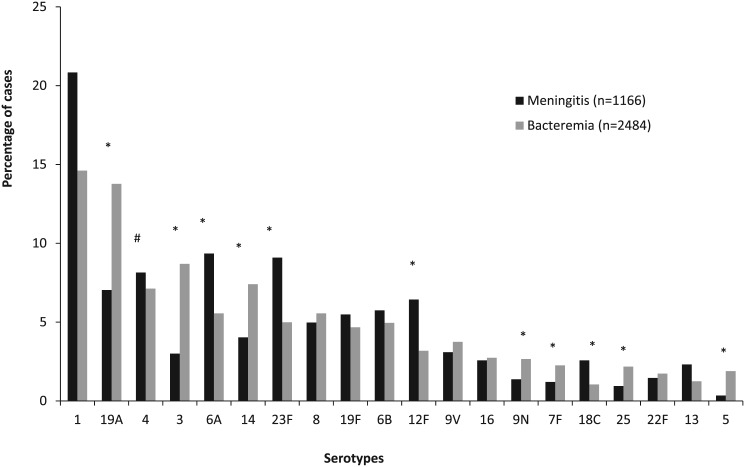
Distribution of pneumococcal serotypes amongst patients aged ≥15 years with invasive pneumococcal disease (IPD) in South Africa from 2003–2008, by clinical syndrome. # Reference group. *Statistically significant at p<0.05

Serotypes 6A (RRR 1.5, 95% CI 1.0–2.1), 23F (RRR 1.6, 95% CI 1.1–2.3), 12F (RRR 1.8, 95% CI 1.2–2.7) and 18C (RRR 2.2, 95% CI 1.2–3.8) were more likely to present as meningitis, while serotypes 19A (RRR 2.2, 95% CI 1.6–3.2), 3 (RRR 3.3, 95% CI 2.1–5.1), 14 (RRR 2.1, 95% CI 1.4–3.2), 9N (RRR 2.2, 95% CI 1.2–4.0), 7F (RRR 2.1, 95% CI 1.1–4.1), 25 (RRR 2.6, 95% CI 1.3–5.3) and 5 (RRR 6.3, 95% CI 2.2–18.0) were more likely to present as bacteremia when compared to serotype 4 (Fig 4).

**Fig 5:**
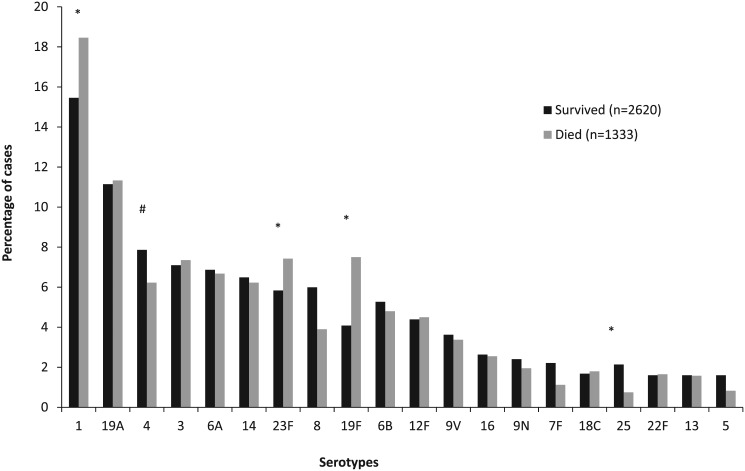
Distribution of pneumococcal serotypes amongst patients aged ≥15 years with invasive pneumococcal disease (IPD) in South Africa from 2003–2008, by in-hospital outcome. # Reference group. *Statistically significant at p<0.05

In addition, serotypes 1 (RRR 1.5, 95% CI 1.1–2.0), 23F (RRR 1.6, 95% CI 1.1–2.3) and 19F (RRR 2.3, 95% CI 1.6–3.4) were significantly more likely to cause death compared to serotype 4. Serotype 25 (RRR 0.4, 95% CI 0.2–0.9) was associated with a lower probability of death compared to serotype 4 (Fig 5).

### Factors associated with in-hospital mortality overall

Among all patients with IPD aged ≥15 years, on multivariable analysis, compared to serotype 4, infection with serotype 1 (OR 1.9, 95% CI 1.1–3.5), 14 (OR 2.1, 95% CI 1.0–4.2) and 19F (OR 2.9, 95% CI 1.4–6.1) was associated with increased in-hospital mortality (Table 2). Additional factors associated with in-hospital mortality on multivariable analysis controlling for province were increasing age (25–44 years OR 1.8 95% CI 1.0–3.0; 45–64 years OR 3.6, 95% CI 2.0–6.4; ≥65 years OR 5.2, 95% CI 1.9–14.1 vs 15–24 years), meningitis (vs bacteremia) (OR 4.1, 95% CI 3.0–5.6), history of antibiotic use in the two months preceding the IPD episode (OR 3.9 95% CI 2.5–6.2), inappropriate antibiotic prescription (OR 2.4, 95% CI 1.7–3.2) and being HIV infected (OR 1.7, 95% CI 1.0–2.8).

**Table 2:** Univariate and multivariable analysis of factors associated with mortality amongst patients aged ≥15 years with invasive pneumococcal disease (IPD) in South Africa, 2003–2008.

Risk factor		Percent of all enrolled cases	Case-fatality ratio	Univariate analysis		Multivariable analysis	
		n/N (%)	n/N (%)	OR (95% CI)	p	OR (95% CI)	p
Age group (years)	15–24	406/3953 (10)	92/406 (23)	Reference	Reference	Reference	Reference
	25–44	2527/3953 (64)	793/2527 (31)	1.6 (1.2–2.0)	<0.001	1.8 (1.0–3.0)	0.037
	45–64	810/3953 (21)	348/810 (43)	2.6 (2.0–3.4)	<0.001	3.6 (2.0–6.4)	<0.001
	≥65	210/3953 (5)	100/210 (48)	3.1 (2.2–4.4)	<0.001	5.2 (1. 9–14.1)	0.001
Province poverty level*	Low	2934/3953 (74)	915/2934 (31)	Reference	Reference	Reference	Reference
	Intermediate	780/3953 (20)	301/780 (39)	1.4 (1.2–1.6)	<0.001	1.6 (1.2–2.4)	0.005
	High	239/3953 (6)	117/239 (49)	2.1 (1.6–2.8)	<0.001	1.7 (1.0–3.0)	0.042
Disease syndrome	Meningitis	1166/3923 (30)	641/1166 (55)	4.0 (1.2–2.2)	<0.001	4.1 (3.0–5.6)	<0.001
	Bacteremic pneumonia	2484/3923 (63)	576/2484 (23)	Reference	Reference	Reference	Reference
	Other	273/3923(7)	98/273 (36)	1.9 (0.3–0.5)	<0.001	1.6 (0.9–2.9)	0.104
Pitt bacteremia score	<4	2993/3953 (76)	864/2993 (29)	Reference	Reference		
	≥4	960/3953 (24)	469/960 (49)	2.4 (2.0–2.7)	<0.001		
Prior antibiotic use (24 hours)	Yes	96/2968 (3)	41/96 (43)	2.1 (1.4–3.1)	0.001		
	No	2872/2968 (97)	759/2872 (26)	Reference	Reference		
Prior antibiotic use (2 months)	Yes	149/2402 (6)	59/149 (40)	2.6 (1.8–3.6)	<0.001	3.9 (2.5–6.2)	<0.001
	No	2253/2402 (94)	458/2253 (20)	Reference	Reference	Reference	Reference
Appropriate antibiotic prescription	Yes	3005/3923 (77)	900/3005 (30)	Reference	Reference	Reference	Reference
	No	918/3923 (23)	415/918 (45)	1.9 (1.7–2.3)	<0.001	2.4 (1.7–3.2)	<0.001
HIV status	Positive	2309/2580 (89)	687/2309 (30)	1.5(1.1–2.0)	0.013	1.7 (1.0–2.8)	0.035
	Negative	271/2580 (11)	61/271 (23)	Reference	Reference	Reference	Reference
Nosocomial infection	Yes	168/3953 (4)	73/168 (44)	1.5 (1.1–2.1)	0.007		
	No	3785/3953(96)	1260/3785 (33)	Reference	Reference		
Multi-drug resistant	Yes	609/3953 (15)	230/609 (38)	1.2 (1.0–1.5)	0.022		
	No	3344 (85)	1103/3344(33)	Reference	Reference		
Serotype	1	651/3953 (17)	246/651 (38)	1.5 (1.1–2.0)	0.007	1.9 (1.1–3.5)	0.034
	19A	443/3953 (11)	151/443 (34)	1.3 (0.9–1.8)	0.128	1.8 (0.9–3.4)	0.075
	4	289/3953 (7)	83/289 (29)	Reference	Reference	Reference	Reference
	3	284/3953 (7)	98/284 (35)	1.3 (0.9–1.9)	0.137	1.2 (0.5–2.5)	0.727
	6A	269/3953 (7)	89/269 (33)	1.2 (0.9–1.8)	0.265	0.8 (0.4–1.6)	0.501
	14	253/3953 (6)	83/253 (33)	1.2 (0.8–1.8)	0.303	2.1 (1.0–4.2)	0.049
	23F	252/3953 (6)	99/252 (39)	1.6 (1.1–2.3)	0.010	1.9 (0.9–3.8)	0.073
	8	209/3953 (5)	52/209 (25)	0.8 (0.6–1.2)	0.342	1.2 (0.5–2.7)	0.637
	19F	207/3953 (5)	100/207 (48)	2.3 (1.6–3.4)	<0.001	2.9 (1.4–6.1)	0.005
	6B	202/3953 (5)	64/202 (32)	1.2 (0.8–1.7)	0.481	1.4 (0.7–2.8)	0.428
	12F	175/3953 (4)	60/175 (34)	1.3 (0.9–1.9)	0.209	1.2 (0.5–2.9)	0.757
	9V	140/3953 (4)	45/140 (32)	1.18 (0.8–1.8)	0.468	1.8 (0.8–4.4)	0.187
	16	103/3953 (3)	34/103 (33)	1.22 (0.8–2.0)	0.414	0.4 (0.1–1.5)	0.201
	9N	89/3953 (2)	26/89 (29)	1.0 (0.6–1.7)	0.928	0.9 (0.3–3.0)	0.888
	7F	73/3953 (2)	15/73 (21)	0.6 (0.3–1.2)	0.163	1.0 (0.3–3.0)	0.965
	18C	68/3953 (2)	24/68 (35)	1.4 (0.8–2.4)	0.288	1.3 (0.4–4.1)	0.611
	25	66/3953 (2)	10/66 (15)	0.4 (0.2–0.9)	0.027	0.8 (0.2–3.1)	0.701
	22F	64/3953 (2)	22/64 (34)	1.3 (0.7–2.3)	0.371	1.3 (0.4–4.7)	0.674
	13	63/3953 (2)	21/63 (33)	1.2 (0.7–2.2)	0.468	2.0 (0.7–5.8)	0.234
	5	53/3953 (1)	11/53 (21)	0.7 (0.3–1.3)	0.235	0.3 (0.0–3.0)	0.291

N-number, OR—Odds ratio, CI—confidence interval, HIV—Human immunodeficiency virus, PCV- pneumococcal conjugate vaccine. Only factors statistically significant on univariate analysis are included in the table. Additional variables evaluated were gender, race, presence of underlying medical conditions other than HIV and penicillin non-susceptibility.

*The low poverty rate group consisted of Gauteng and Western Cape. The intermediate poverty rate group consisted of KwaZulu-Natal, Free State, Northern Cape and North West. The high poverty rate group consisted of Eastern Cape, Mpumalanga and Limpopo.

### Factors associated with in-hospital mortality stratified by clinical syndrome

Among patients with bacteremic pneumonia, on multivariable analysis, compared to serotype 4, infection with serotype 14 (OR 2.2, 95% CI 1.1–4.7) and 19F (OR 3.5, 95% CI 1.6–8.0) was associated with increased in-hospital mortality (Table 3). Additional factors associated with in-hospital mortality on multivariable analysis among patients with bacteremic pneumonia were increasing age group (25–44 years OR 2.7 95% CI 1.5–5.0; 45–64 years OR 6.2, 95% CI 3.1–12.7; ≥65 years OR 11.7, 95% CI 3.4–40.4 vs 15–24 years), history of antibiotic use in the 2 months preceding the IPD episode (OR 2.3 95% CI 1.4–3.9) and inappropriate antibiotic prescription (OR 2.8, 95% CI 2.0–3.8).

**Table 3:** Multivariable analysis of factors associated with in-hospital mortality amongst patients aged ≥15 years with invasive pneumococcal disease (IPD) in South Africa from 2003–2008, with bacteremic pneumonia and meningitis separately.

Risk factor		Bacteremic pneumonia			Meningitis		
		Case-fatality ratio n/N (%)	Adjusted odds ratio (95% confidence interval)	p	Case-fatality ratio n/N (%)	Adjusted odds ratio (95% confidence interval)	p
Age group (years)	15–24	23/225 (10)	Reference	Reference	54/141 (38)	Reference	Reference
	25–44	320/1582 (20)	2.7 (1.5–5.0)	0.009	421/770 (55)	1.8 (0.9–4.1)	0.110
	45–64	175/527 (33)	6.2 (3.1–12.7)	<0.001	145/226 (64)	7.1 (2.8–18.2)	<0.001
	≥65	58/150 (39)	11.7 (3.4–40.4)	<0.001	21/29 (72)	8.3 (0.6–108.4)	0.106
Pitt bacteremia score	<4				401/807 (50)	Reference	
	≥4				240/359 (67)	2.4 (1.4–4.1)	<0.001
Prior antibiotic use (2 months)	Yes	27/95 (28)	2.3 (1.4–3.9)	0.001	22/34 (65)	5.0 (2.0–12.5)	<0.001
	No	200/1519 (13)	Reference	Reference	220/577 (38)	Reference	Reference
Appropriate antibiotic prescription	Yes	397/1976 (20)	Reference	Reference			
	No	179/508 (35)	2.8 (2.0–3.8)	<0.001			
HIV status	Positive				336/634 (53)	6.1 (2.1–17.6)	0.001
	Negative				19/73 (26)	Reference	Reference
Serotype	1	60/363 (17)	1.2 (0.6–2.4)	0.709	174/243 (72)	2.0 (0.8–4.6)	0.149
	19A	93/342 (27)	1.5 (0.8–3.1)	0.251	52/82 (63)	1.1 (0.4–3.2)	0.811
	4	29/177 (16)	Reference	Reference	51/95 (54)	Reference	Reference
	3	69/216 (32)	2.0 (1.0–4.2)	0.061	13/35 (37)	0.2 (0.02–1.5)	0.103
	6A	31/138 (22)	0.7 (1.3–2.0)	0.517	50/109 (46)	0.5 (0.2–1.2)	0.125
	14	55/184 (30)	2.2 (1.1–4.7)	0.039	20/47 (43)	0.5 (0.1–1.8)	0.267
	23F	27/124 (22)	1.5 (0.6–3.7)	0.352	59/106 (56)	0.9 (0.2–2.9)	0.765
	8	14/138 (10)	0.8 (0.3–2.0)	0.649	32/528 (55)	0.9 (0.3–3.1)	0.851
	19F	51/116 (44)	3.5 (1.6–8.0)	0.002	35/64 (55)	0.7 (0.2–2.5)	0.567
	6B	22/123 (18)	1.0 (0.4–2.5)	0.954	38/67 (57)	1.0 (0.4–3.0)	0.975
	12F	16/79 (20)	1.2 (0.4–3.3)	0.778	38/75 (51)	0.7 (0.2–2.6)	0.598
	9V	23/93 (25)	2.0 (0.8–5.2)	0.153	19/36 (53)	0.2 (0.01–1.4)	0.230
	16	19/68 (28)	0.6 (0.2–2.4)	0.508	12/30 (40)	0.2 (0.03–0.9)	0.040
	9N	17/66 (26)	1.1 (0.4–3.5)	0.863	6/16 (38)	0.4 (0.1–4.0)	0.444
	7F	10/56 (18)	0.9 (0.2–3.5)	0.868	3/14 (21)	0.4 (0.1–2.1)	0.271
	18C	6/26 (23)	1.5 (0.4–85.8)	0.583	10/30 (47)	1.4 (0.3–7.0)	0.716
	25	7/54 (13)	0.5 (0.1–2.6)	0.440	3/11 (27)	0.2 (0.01–2.0)	0.155
	22F	11/43 (26)	1.6 (0.4–5.6)	0.498	9/17 (53)	0.5 (0.1–3.0)	0.409
	13	7/31 (23)	1.6 (0.5–5.7)	0.435	11/27 (41)	1.1 (0.3–5.2)	0.851
	5	9/47 (19)	0.7 (0.2–3.6)	0.703	2/4 (50)	0.8 (0.02–36.0)	0.923

N-number, OR—Odds ratio, CI—confidence interval, HIV—Human immunodeficiency virus. Serotype was retained in the multivariate models a priori because it was the main variable of interest. Only factors statistically significant on multivariable analysis for the syndrome of interest are presented in the table. Additional factors evaluated for patients with bacteremic pneumonia were: (a) factors non-significant on univariate analysis: race, gender, province poverty level, appropriate antibiotic prescription (b) factors significant on univariate analysis but not on multivariable analysis: underlying medical conditions other than HIV, serotype, receipt of antibiotics in the 24 hours preceding culture, nosocomial infection, penicillin non-susceptibility, multidrug resistance. Additional factors evaluated for patients with meningitis were: (a) factors non-significant on univariate analysis: gender, province poverty level, receipt of antibiotics in the 24 hours preceding culture, appropriate antibiotic prescription, nosocomial infection, penicillin non-susceptibility, multidrug resistance (b) factors significant on univariate analysis but not on multivariable analysis: race, underlying medical conditions other than HIV, serotype.

Among patients with meningitis, on multivariable analysis, compared to serotype 4, infection with serotype 16 (OR 0.2, 95% CI 0.03–0.9) was associated with lower in-hospital mortality (Table 3). Additional factors associated with in-hospital mortality on multivariable analysis among patients with meningitis were age group 45–64 years (OR 7.1, 95% CI 2.8–18.2 vs 15–24 years), Pitt bacteremia score ≥4 (OR 2.4, 95% CI 1.4–4.1), history of antibiotic use in the 2 months preceding the IPD episode (OR 5.0 95% CI 2.0–12.5) and being HIV infected (OR 6.1, 95% CI 2.1–17.6).

### Factors associated with in-hospital mortality stratified by HIV-infection status

Amongst HIV-infected individuals, on multivariable analysis, compared to serotype 4, infection with serotype 19F (OR 2.6, 95% CI 1.2–5.7) was associated with increased in-hospital mortality (Table 4). Additional factors associated with in-hospital mortality on multivariable analysis among HIV-infected individuals included increasing age group (25–44 years OR 1.9, 95% CI 1.1–3.4; 45–64 years OR 3.6, 95% CI 1.9–68; ≥65 years OR 6.9, 95% CI 1.8–25.9 vs 15–24 years), history of antibiotic use in the 2 months preceding the IPD episode (OR 4.3 95% CI 2.7–7.1), inappropriate antibiotic prescription (OR 2.5, 95% CI 1.8–3.4) and meningitis (OR 4.6 95% CI 3.3–6.0 vs bacteremia). CD4+ T cell count was available for only 1315 of 2309 (57%) HIV-infected individuals, of whom 1031 (45%) were <200 cells/mm^3^. CFRs were higher in patients with CD4+ T cell count <200 (297/1031, 29%) compared to those with CD4+ T cell count ≥200 cells/mm^3^ (38/284, 13%, p<0.001). CD4 + T cell count <200 cells/mm^3^ was not associated with serotype (p = 0.754). Amongst 1744 HIV-infected individuals with available data, 10% (n = 178) reported receiving anti-retroviral therapy (ART). CFRs were similar in individuals receiving (47/178, 26%) and not receiving (354/1566, 23%) ART (p = 0.254).

**Table 4:** Multivariable analysis of factors associated with in-hospital mortality amongst patients aged ≥15 years with invasive pneumococcal disease (IPD) in South Africa from 2003–2008, for HIV-infected and—uninfected patients separately.

Risk factor		HIV-infected			HIV-uninfected		
		Case-fatality ratio n/N (%)	Adjusted odds ratio (95% confidence interval)	p	Case-fatality ratio n/N (%)	Adjusted odds ratio (95% confidence interval)	p
Age group (years)	15–24	48/210 (23)	Reference		4/44 (9)	Reference	
	25–44	482/1688 (29)	1.9 (1.1–3.4)	0.025	25/118 (21)	4.7 (1.4–16.4)	0.015
	45–64	145/384 (38)	3.6 (1.9–6.8)	<0.001	24/84 (29)	11.8 (3.1–44.6)	<0.001
	≥65	11/27 (41)	6.9 (1.8–25.9)	0.004	8/25 (32)	12.9 (2.6–64.6)	0.002
Race	Black				55/214 (26)	Reference	
	Other				6/56 (11)	0.2 (0.1–0.5)	0.002
Pitt bacteremia score	<4				37/211 (18)	Reference	
	≥4				24/60 (40)	3.9 (1.8–8.6)	0.001
Province poverty level*	Low	517/1855 (28)	Reference				
	Intermediate	129/357 (36)	1.9 (1.3–2.7)	0.001			
	High	41/97 (42)	1.9 (1.1–3.3)	0.025			
Prior antibiotic use (2 months)	Yes	43/95 (45)	4.3 (2.7–7.1)	<0.001			
	No	232/1383 (17)	Reference				
Appropriate antibiotic prescription	Yes	469/1782 (26)	2.5 (1.8–3.4)	<0.001			
	No	213/515 (41)	Reference				
Disease syndrome	Meningitis	336/634 (53)	4.6 (3.3–6.0)	<0.001	19/73 (26)	1.6 (0.7–3.7)	0.226
	Bacteremic pneumonia	317/1550 (20)	Reference		34/170 (20)	Reference	
	Other	29/113 (26)	1.6 (0.8–3.0)	0.001	7/27 (26)	1.7 (0.6–5.3)	0.349
Serotype	1	104/317 (33)	1.7 (0.9–3.2)	0.104	20/78 (26)	5.0 (0.6–44.6)	0.151
	19A	91/283 (32)	1.7 (0.9–3.4)	0.098	5/22 (23)	6.2 (0.6–65.5)	0.132
	4	45/178 (25)	Reference		2/22 (9)	Reference	
	3	30/149 (20)	0.8 (0.3–1.9)	0.480	7/24 (29)	7.2 (0.7–73.7)	0.095
	6A	51/174 (29)	0.6 (0.3–1.3)	0.200	5/16 (32)	6.5 (0.6–72.1)	0.128
	14	44/151 (29)	2.1 (1.0–4.4)	0.052	2/16 (13)	2.5 (0.2–34.4)	0.483
	23F	69/164 (42)	1.6 (0.8–3.2)	0.898	3/11 (27)	7.9 (0.6–103.2)	0.116
	8	23/112 (21)	1.1 (0.5–2.6)	0.726	4/21 (19)	3.5 (0.3–40.1)	0.317
	19F	53/118 (45)	2.6 (1.2–5.7)	0.014	3/9 (33)	12.3 (0.9–162.2)	0.056
	6B	36/126 (29)	1.2 (0.6–2.6)	0.994	1/13 (8)	2.4 (0.1–45.9)	0.561
	12F	30/101 (30)	1.0 (0.5–1.9)	0.907	2/4 (50)	23.9 (1.2–476.5)	0.037
	9V	30/97 (31)	1.7 (0.7–4.1)	0.257	0/1 (0)	Omitted	
	16	16/63 (25)	0.3 (0.1–1.1)	0.060	3/7 (43)	28.3 (1.9–414.9)	0.015
	9N	13/54 (24)	0.7 (0.2–2.7)	0.716	2/7 (29)	16.3 (1.1–255.8)	0.047
	7F	12/49 (24)	0.9 (0.3–2.9)	0.300	0/4 (0)	Omitted	
	18C	12/38 (32)	1.2 (0.4–3.7)	0.828	1/3 (33)	7.5 (0.3–198.6)	0.226
	25	6/43 (14)	0.7 (0.2–2.9)	0.645	0/1 (0)	Omitted	
	22F	10/38 (26)	1.3 (0.3–4.6)	0.736	0/2 (0)	Omitted	
	13	9/29 (31)	2.2 (0.7–6.8)	0.785	1/5 (20)	2.6 (0.1–78.6)	0.581
	5	3/25 (12)	0.2 (0.1–2.9)	0.267	0/5 (0)	Omitted	

N-number, OR—Odds ratio, CI—confidence interval, HIV—Human immunodeficiency virus. Only factors statistically significant on multivariable analysis for the syndrome of interest are presented in the table. Serotype was retained in the multivariate models a priori because it was the main variable of interest, and clinical diagnosis because it is an important potential confounder of the association between serotype and mortality.

Amongst HIV-uninfected individuals, on multivariable analysis, compared to serotype 4, infection with serotype 12F (OR 23.9, 95% CI 1.2–176.5), serotype 16 (OR 28.3 95% CI 1.9–414.9) and serotype 9N (OR 16.3 95% CI 1.1–255.8) was associated with increased in-hospital mortality. Additional factors associated with in-hospital mortality among HIV-uninfected individuals included increasing age group (25–44 years OR 4.7, 95% CI 1.4–16.4; 45–64 years OR 11.8, 95% CI 3.1–44.6; ≥65 years OR 12.9, 95% CI 2.6–64.6 vs 15–24 years) and elevated Pitt bacteraemia score (OR 3.9, 95% CI 1.8–8.6).

Individuals aged 15–17 years made up only 1% (51/3953) of included individuals. Sensitivity analysis excluding individuals aged 15–17 years did not change any of the study conclusions (data not shown).

## Discussion

We have described extremely high in-hospital mortality in individuals aged ≥15 years with IPD in South Africa, particularly for meningitis, where CFRs exceed 50%. Individual serotypes (1, 14 and 19F) were associated with increased mortality, even after controlling for confounders, suggesting pneumococcal capsule characteristics may be related to virulence. Other important factors associated with increased in-hospital mortality were increasing age and underlying HIV infection. All of the serotypes associated with increased mortality are included in PCV-10 and -13 and use of these vaccines in infants has reduced the burden of these serotypes in children as well as among adults through a herd effect [25, 26].

As has been described in other studies, serotypes causing IPD varied by age group, HIV status and clinical syndrome, all important factors potentially associated with mortality[27, 28]. For this reason, it is important to control for potential confounders when assessing the association between serotype and mortality. We found serotypes 1, 14 and 19F to be significantly associated with mortality, and particularly when presenting with bacteremia. Previous studies have found serotype 19F to be associated with increased CFR [11, 12, 29]. Serotype 14 was also associated with increased mortality in the current study, which has not been previously described. Serotype 1 was the commonest serotype in our study population and we found it to be associated with increased CFR. Some studies have found serotype 1 to be associated with decreased risk of death [11, 12], although increased CFR as found in our study has been observed previously [30]. Previous studies have suggested that serotypes with increased relative risks of death had a higher carriage prevalence and low invasiveness[11]. Serotype 1 has been described as among the more invasive serotypes and serotype 19F among the less invasive serotypes [12, 29, 31, 32].

Differences in the association between serotype and mortality in our study compared to previous studies could be partly as a result of the high HIV-prevalence (89% HIV prevalence amongst included IPD cases) in the population studied. Interestingly, when stratified by HIV-infection status, different serotypes were found to be associated with increased mortality in HIV-infected (serotype 19F) and HIV-uninfected (serotypes 12F, 16 and 9N) individuals. Serotype 1 was not found to be independently associated with increased mortality in either the HIV-infected or -uninfected group, although CFRs were elevated for this serotype in both HIV-infected and—uninfected individuals. Other factors associated with mortality also differed between the two groups. In the HIV-infected subgroup, meningitis was associated with almost five times increased odds of death, but mortality was not significantly elevated in patients with meningitis in the HIV-uninfected group. The overall CFR was extremely high (55%) in patients with meningitis and elevated compared to individuals with bacteremic pneumonia. A study from Malawi, a country with a high population HIV prevalence, found 65% mortality in patients with meningitis compared to 20% for bacteremia[33]. In HIV-infected individuals consumption of antibiotics in the two months preceding hospitalization was predictive of elevated mortality; this likely reflected the severity of underlying HIV-related illness, rather than infection with antibiotic non-susceptible organisms, because strain susceptibility was not related to mortality on multivariable analysis. Residence in intermediate and high poverty level provinces was also associated with increased mortality in HIV infected individuals. This is possibly related to different specimen-taking practices[34], where in less-resourced provinces only the sickest individuals have specimens collected for culture; or due to worse access to care or quality of care, although we do not have data to support this hypothesis.

We found HIV-infected individuals to have an overall two times greater odds ratio of in-hospital death compared to HIV-uninfected individuals. When stratified by syndrome, this association was only seen in patients with meningitis (who experience six times increased odds of death) but not in patients with bacteremic pneumonia. This difference in mortality association by clinical syndrome is similar to what was found in a study of IPD in patients aged <15 years from South Africa in the pre-PCV era[35]. Published data on the association between HIV infection and mortality vary, with some studies describing increased mortality in HIV-infected individuals and others not finding this association[36–39]. These varying findings could result from different populations studied including differing clinical syndromes included, different proportions of patients receiving ART as well as limited statistical power to detect an association in some studies.

Studies of the association between serotype and mortality have varied in the patient populations included, with some studies including only patients with bacteremic pneumonia and others including all IPD[11, 29, 40]. When stratified by clinical syndrome, amongst patients with bacteremic pneumococcal pneumonia, serotype 19F remained associated with increased mortality, while no serotype was associated with increased mortality in patients with meningitis. This suggests that the association between serotype and mortality may vary depending on the clinical syndrome evaluated.

Other factors associated with increased mortality included increasing age group, which has been described in several studies, most likely as a result of immunosenescence[41–44]. Notably, the numbers of cases aged ≥65 years was low, possibly as a result of reduced health seeking in this age group as well as the relatively younger population than many high income countries and the relatively low life expectancy in South Africa[45, 46]. In contrast to previous studies, we did not find underlying illnesses (other than HIV) to be associated with increased in-hospital mortality. This could reflect poor diagnosis and documentation of underlying illness data in our setting or reflect the relatively smaller contribution of underlying illness to in-hospital outcome in this predominantly HIV-infected population[5–7].

Our study had some limitations. Only patients admitted to enhanced surveillance sites with viable isolates were included. Patients at enhanced sites differed from those presenting to non-enhanced sites with regard to a number of characteristics and this could potentially have introduced bias. Because of the nature of laboratory-based surveillance, only patients who presented to a healthcare facility and who had specimens taken could be included; these individuals may not be fully representative, especially in the poorer provinces. We examined a large number of serotypes individually, which may have reduced our power to detect significant associations, however we did have data on almost 4000 individuals and >1000 deaths. The ability to detect a significant association with mortality for different serotypes could have been affected by available numbers of cases allowing insufficient power to detect statistically significant associations in some cases. Some previous studies did not assess severity association for individual serotypes but rather grouped them, limiting the ability to compare with our findings [40]. We elected not to group serotypes for the mortality analysis as several different grouping have been used in the literature, and all groupings are somewhat arbitrary and could introduce spurious associations[11, 29, 40]. We included individuals aged ≥15 years in our study, although a cut-off of ≥18 years is more standard for adults. We did this because in South Africa 15 years is the age at which patients are generally admitted to adult rather than pediatric wards in South Africa. Importantly, individuals aged 15–17 years only made up 1% of the study population and study conclusions remained unchanged on sensitivity analysis excluding this group of individuals. CD4+ T cell count data was only available for 56% of HIV-infected patients and we therefore did not include it in the analysis of factors associated with mortality; CD4+ T cell count, however, was not associated with serotype and it is therefore unlikely to be a confounder of the association between serotype and mortality.

In conclusion, we have found that individual serotypes, included in PCV-13, were associated with increasing mortality in the pre-PCV era in South Africa, even controlling for potential confounding. HIV infection was also an important risk factor for death in our study, especially among patients with meningitis and older individuals. Studies of the association of pneumococcal serotype and mortality, following widespread PCV introduction and possible serotype replacement, may be informative.
